# Genetic studies of Crohn's disease: Past, present and future

**DOI:** 10.1016/j.bpg.2014.04.009

**Published:** 2014-06

**Authors:** Jimmy Z. Liu, Carl A. Anderson

**Affiliations:** The Wellcome Trust Sanger Institute, Hinxton CB10 1SA, UK

**Keywords:** Crohn's, Genetics, Genomics, Genotyping, Sequencing

## Abstract

The exact aetiology of Crohn's disease is unknown, though it is clear from early epidemiological studies that a combination of genetic and environmental risk factors contributes to an individual's disease susceptibility. Here, we review the history of gene-mapping studies of Crohn's disease, from the linkage-based studies that first implicated the *NOD2* locus, through to modern-day genome-wide association studies that have discovered over 140 loci associated with Crohn's disease and yielded novel insights into the biological pathways underlying pathogenesis. We describe on-going and future gene-mapping studies that utilise next generation sequencing technology to pinpoint causal variants and identify rare genetic variation underlying Crohn's disease risk. We comment on the utility of genetic markers for predicting an individual's disease risk and discuss their potential for identifying novel drug targets and influencing disease management. Finally, we describe how these studies have shaped and continue to shape our understanding of the genetic architecture of Crohn's disease.

## Introduction

Crohn's disease is a chronic inflammatory disease of the gastrointestinal tract affecting 26–200 per 100,000 in European populations [1]. Along with ulcerative colitis, it is one of the two major forms of inflammatory bowel disease (IBD). The exact causes of Crohn's disease are unknown, though it is likely to involve a disrupted immunological response to gut microbiota in genetically susceptible individuals [2]. There is currently no known cure and disease is managed by a combination of immune-suppressing medications, dietary changes or surgery.

## Family and twin studies

Epidemiological observations in the 1930s first suggested an inherited component to Crohn's disease risk. Familial clustering showed that 2–14% of patients have a family history of CD [3], while estimates of the sibling relative risk ratio (the ratio of disease risk among siblings of patients compared with that in the general population, i.e. the population prevalence) ranged from 15–42 [3]. The variation in these estimates highlights the difficulty in obtaining accurate heritability measures for relatively rare diseases such as Crohn's. Confounders also include inconsistent study design (e.g. only counting first degree relatives rather than all relatives), sample selection bias (e.g. using hospital cases that are likely to have a more severe form of the disease than the general Crohn's population), and variation in disease prevalence rates, both between different populations and over time [3–7]. Moreover, these observations do not in themselves suggest a role for genetics in disease risk because familial resemblance can also be a consequence of shared environmental factors.

Twin studies have now provided compelling evidence for a significant genetic component to Crohn's disease risk. The twin design assumes that the environmental component to phenotypic variation is the same between monozygotic (MZ) and dizygotic (DZ) twins, and thus the difference in disease concordance rates between sets of MZ and DZ twin pairs can be used to estimate the additive genetic, shared environmental and unique environmental components of disease risk. A meta-analysis of six twin studies with a combined set of 112 MZ and 196 DZ twin pairs reported concordance rates of 30.3% and 3.6% respectively [8], indicating that a large component of Crohn's disease risk is indeed genetic. Together, these family and twin studies provided the motivation for the first wave of gene-mapping studies throughout the mid-1990s aimed at identifying the regions of the genome that contribute to Crohn's disease risk.

## Linkage studies

A linkage study identifies regions of the human genome underlying disease susceptibility by testing a series of marker alleles for cosegregation (linkage) with disease status across a number of families (or a single large family with multiple affected members). Owing to the large size of chromosomal segments segregating within a typical family, around 300 evenly distributed microsatellite markers are usually sufficient to capture the majority of positions where the chromosomes of the parents crossed over during meiosis (recombination events). The evidence for linkage in a region is evaluated by metrics such as a LOD (logarithm of odds) score, which compares the probability that the genotyped marker and the hypothetical disease locus are inherited together in the observed data versus the probability of observing the cosegregation pattern purely by chance. A typical linkage study will report all loci with LOD scores greater than three, which corresponds to the data being 1000 times more likely to arise due to cosegregation with disease than by chance [9]. By the mid-nineties, linkage studies had proven to be a robust means of identifying highly penetrant loci underlying monogenic disease such as cystic fibrosis [10] and Huntington's disease [11] and the utility of the method for mapping complex disease loci was increasingly being explored. From 1996 to 2004, 11 linkage studies were performed for Crohn's disease (reviewed here [7]), the largest of which was a meta-analysis consisting of 1068 affected relative pairs [12].

The first Crohn's disease linkage study in 1996 identified a significant disease susceptibility locus on chromosome 16 (dubbed IBD1) [13]. This result was confirmed in subsequent studies [14–20] and in 2001 the specific causal mutations that underlie risk were localised to three low frequency coding variants (R702W, G908R and L1007fs) within the *NOD2* gene (at that time, also known as *CARD15*) [21–25]. These three variants individually had odds ratios (ORs) of 2–4 in heterozygotes and 20–40 for homozygotes, and at least one mutation was present in 30–40% of Crohn's disease cases compared with 6–7% in European controls [7].

Spurred on by the discovery of *NOD2*, additional linkage studies of Crohn's disease (and other common complex diseases) were undertaken; The results of these studies were largely disappointing, with few loci being consistently replicated [7]. This lack of success suggested that complex diseases, in contrast to Mendelian diseases, were unlikely to be driven by the highly penetrant risk loci that linkage is well powered to detect. In 1996 a seminal paper was published in Science proposing that complex diseases are underpinned by common variants of modest effect [26]. The authors demonstrated that, for a risk allele of 50% frequency and OR of 1.5, around 18,000 affected sib-pairs would be needed to detect the locus via linkage. In contrast, they reported that less than 1000 trios would be needed to detect such a locus adopting the transmission/disequilibrium association test of Spielman et al. [27]. Technological limitations at the time restricted the immediate uptake of the association study design; such studies require that a causal variant (or another variant in high linkage disequilibrium (LD) to the causal variant) is directly genotyped in order to detect a significant signal of association.

## Candidate gene association studies

While it was infeasible to test for association at markers across the entire genome, markers within individual genes were often tested for association. Genes were selected based on *a priori* knowledge of biological function or because the lay within a region implicated through linkage analysis. These candidate gene studies typically involved genotyping a set of markers within a gene of interest in a sample of disease cases and controls, and testing for statistically significant differences in allele frequencies between the two groups. Other study designs such as transmission disequilibrium tests in parent-offspring trios were also often used.

Results from the majority of candidate gene studies of Crohn's disease were disappointing, with initial findings often failing to replicate in subsequent studies [28–31]. A combination of small sample sizes, false-positive association, publication bias and failure to account for multiple comparisons meant that as many as 95% of findings from candidate gene studies of complex traits during this era were false [32,33]. In some cases, the lack of power in these studies meant that variants in genes that later became established risk loci were missed altogether (for instance, *IL10* [34,35]). Ultimately however, it would take a combination of technological advances and a greater appreciation of the need for much larger sample sizes to make the identification of bona fide risk loci routine.

## Genome-wide association studies

In the early 2000s, along with the closing phases of Human Genome Project, concurrent efforts were underway to gauge the extent of human genetic variation at the population level. Projects such as the SNP Consortium and dbSNP had catalogued over 1.4 million single nucleotide polymorphisms (SNPs) by 2001 [36,37]. It was found that common SNPs in physical proximity formed LD blocks punctuated by hotspots of recombination [38]. These correlation patterns were further characterised through the International Hapmap Project, which by 2007 had identified a further 3.1 million SNPs across 270 individuals from three distinct ancestry groups [39]. At the same time, technological advances in microarray technologies made possible the cost-effective genotyping of hundreds of thousands of SNPs spread throughout the genome [40]. The patterns of LD meant that these arrays could effectively survey the majority of common genetic variation in a population by directly genotyping only a fraction of the total number of variants in the genome. In Europeans and East Asians, around five million common SNPs (those with minor allele frequency greater than 5%) can be almost entirely tagged by a selection of approximately 500,000 SNPs [41,42]. Together, these advances paved the way for researchers to perform genome-wide association studies (GWAS) in order to identify loci associated with complex traits or disease risk.

**Fig. 1:**
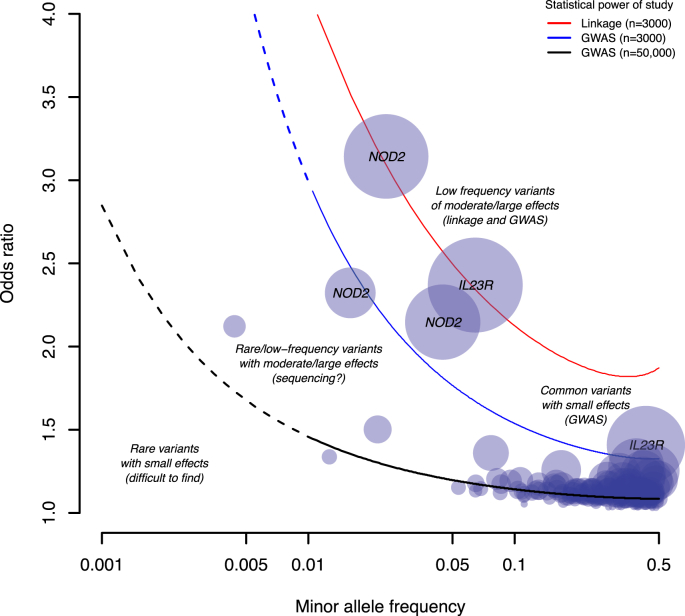
**The genetic architecture of Crohn's disease**. Known Crohn's disease risk variants are plotted according to their minor allele frequency and odds ratio (OR) [59]. The size of the circles represents the amount of variance in Crohn's disease liability explained by that variant. The red, blue and black lines represent the minimum OR and allele frequency for a locus for which a linkage study with 3000 individuals, GWAS with 3000 individuals and GWAS with 50,000 individuals respectively will have >80% statistical power to detect [26,108]. *P*-value thresholds for power calculations were set to *P* < 10^−4^ for linkage and *P* <5 × 10^−8^ for GWAS. The dashed lines represents the allele frequency spectrum of variants that are typically poorly captured on GWAS microarrays (minor allele frequencies less than 1%).

Genome-wide association studies typically look for statistically significant differences in allele (or genotype) frequencies between a large number of diseased individuals and population controls across hundreds of thousands of SNPs spread throughout the genome. The SNPs that show significant association with disease status point to regions of the genome likely to harbour disease relevant genes. Unlike linkage studies, GWAS are not restricted to sibling pairs and families, and also have generally greater statistical power to detect associated loci of small to moderate effect sizes (Fig. 1) [26]. Due to patterns of LD, SNPs that are associated with disease are unlikely to be the true causal variant, but rather are correlated with (‘tag’) an untyped causal variant. In addition, genotypes at SNPs that were not directed assayed can be inferred through imputation algorithms [43,44] based on the genotypes from a representative reference set of haplotypes [39,45,46], allowing for individual studies using different genotyping platforms to be effectively combined into meta-analyses.

The first Crohn's disease GWAS was conducted in a Japanese population in 2005, and identified *TNFSF15* as a susceptibility locus [47]. This was followed by a rush of studies from 2006 to 2008 [48–55], each including approximately 500–2000 Crohn's disease cases and a similar number of controls genotyped at 100,000–600,000 SNPs. Unlike linkage studies, the development of standardised quality control protocols along with strict statistical criteria for claiming association and replication [55,56] meant the vast majority of SNPs that achieved genome-wide statistical significance (association *p*-value <5 × 10^−8^) were true positives. The genes and pathways identified by these early GWAS provided many insights into the biological processes underlying Crohn's disease. Most notably, associations at *ATG16L1* and *IRGM* first suggested a role for autophagy in disease pathogenesis [2,48,52]. Other genes involved in both the innate (*TLR4*, *CARD9*, *IL23R*, *STAT3*) and adaptive immune system (*HLA*, *TNFSF15*, *IRF5*, *PTPN22*) pathways were also implicated [57]. GWAS have also shed light on the genetic overlap between Crohn's and other immune-related diseases. Around 30% of associated variants in these initial studies were shared with ulcerative colitis, while close to 50% of loci are shared with at least one other immune-mediated disease such as type 1 diabetes, coeliac disease or rheumatoid arthritis [58]. Unlike many of these diseases, genes in the human leucocyte antigen (HLA) region only confer a modest effect on Crohn's disease risk (ORs 1.1–1.2). This is in contrast to ulcerative colitis, where several variants in *HLA-B* make the largest contribution to genetic risk (ORs 1.4–1.5) [59].

These early GWAS showed that, with the exception of *NOD2*, the typical effect size of a Crohn's susceptibility locus was modest (OR < 1.3), such that the loci identified only explain a fraction of the known genetic component of Crohn's disease risk (highlighting the ‘missing heritability problem’ [60,61]). While it is likely that a proportion of this missing heritability is due to rare (minor allele frequency less than 1%) and structural variants that are not well-captured on the current generation of GWAS microarrays, a substantial number of common variants will have even smaller effects than those identified, requiring much larger sample sizes to detect [62]. Indeed, for Crohn's disease, it has been estimated that 22% of the differences in individual disease risk seen in the population (variance in disease liability) can be explained by common variants tagged on microarrays [63] – more than double that explained by known risk loci [64].

**Fig. 2:**
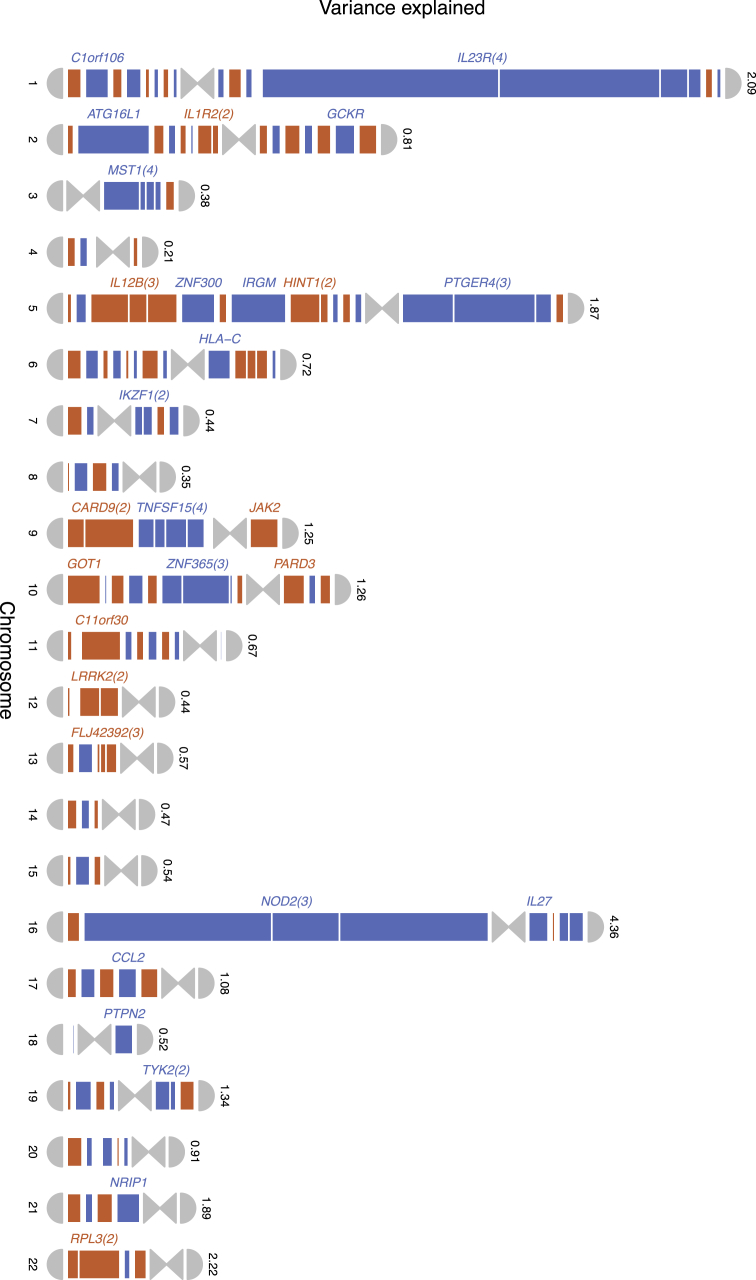
**The Crohn's disease genome**. Known Crohn's disease risk loci are shown according to their location on the long or short arms of chromosomes. The size of each locus indicates the proportion of variance in Crohn's disease liability explained by that locus [59]. Several notable genes are marked. Parentheses next to gene names denote the number of independent risk variants within the locus. The number above each chromosome is the ratio of the total amount of variance explained by that chromosome vs the expected number given the chromosome's size. Sex chromosomes were excluded as no loci have been conclusively implicated, largely due to these chromosomes being overlooked from most GWAS.

An appreciation of the need for larger sample sizes led to the creation of the International IBD Genetics Consortium (IIBDGC) (http://www.ibdgenetics.org/) to bring together investigators and GWAS datasets from IBD genetics groups around the world. From 2008 to 2012 the IIBDGC published three genome-wide association study meta-analyses [59,64,65]. The first of these in 2008 combined data for ∼13,000 individuals from three previously published GWAS and identified 21 new Crohn's susceptibility loci [64]. This was followed two years later by a meta-analysis of six GWAS with a total sample size of ∼50,000 individuals where 30 new loci were identified, bringing the total count to 71 [65]. The most recent meta-analysis in 2012 included 75,000 individuals and doubled the number of known Crohn's susceptibility loci to 140 (Fig. 2) [59]. Along with the 23 loci associated with ulcerative colitis, the total number of 163 inflammatory bowel disease associated loci represents the most for any complex disease to date. These loci were enriched for genes involved in primary immunodeficiencies and this enrichment was even more striking for genes harbouring Mendelian susceptibility to mycobacterial disease (MSMD) risk variants, where six of the eight genes linked to MSMD overlap with IBD. Similarly, seven of the eight genes known to be associated with leprosy are also shared with IBD, and altogether, 66 IBD loci are shared with other immune-mediated diseases. These overlaps suggest that selection pressures driven by mycobacterial infection may have shaped the genetic architecture of Crohn's disease.

**Fig. 3:**
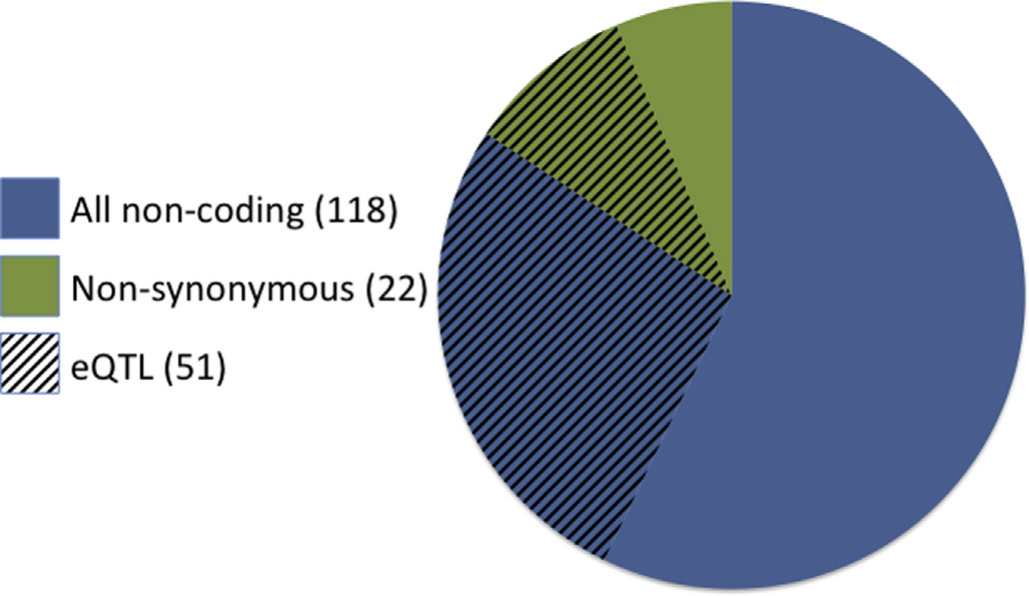
**Proportion of noncoding, nonsynonymous and known eQTLs among known 140 Crohn's disease risk loci**[59]. A locus is labelled as nonsynonymous if the lead SNP is in high linkage disequilibrium (LD; *r*^2^ > 0.8) with a nonsense or missense mutation. Similarly, loci with eQTLs were marked if the lead SNP is in high LD with a known eQTL in studies of liver, brain, fibroblasts, monocytes, T cells and lymphoblastoid cell lines [66,67].

The results of Jostins et al. [59] also highlighted the role of noncoding variation in disease risk. Many of these variants are likely to affect the amount that a gene is expressed (gene regulation) rather than code the protein product. Only nine of the 140 associated loci exclusively harbour variants within coding regions of genes (*IL23R*, *GPR35*, *CD6*, *MUC19*, *GPR65*, *ZNF831*, *ADAM30*, *NOD2*, *FUT2*) while an additional 13 have variants encompassing both coding and noncoding regions (*UBQLN4*, *ITLN1*, *FCGRA2A*, *MST1/BSN*, *SLC22A4*, *REV3L*, *CARD9*, *ZPBP2/GSDMB*, *TUBD1*, *CD226*, *YDJC*, *ATG16L1*, *LACC1*). Altogether, 51 loci overlap variants with a known effect on gene expression (eQTLs – expression quantitative trait loci; Fig. 3). Most studies that detect eQTLs have been limited to only a few hundred individuals in only a small number of cell types (liver, brain, fibroblasts, monocytes, T cells and lymphoblastoid cell lines) [66,67]. The overlap between eQTLs and disease-associated variants will increase as more eQTL studies are performed across larger samples sizes and across different cell types, especially those involved in the immune system in the case of Crohn's disease.

## Targeted genotype arrays

A feature of the latest IIBDGC meta-analysis was the use of the Immunochip custom genotyping array for replicating signals identified in the original GWAS meta-analysis. The Immunochip was designed after the first wave of GWAS meta-analyses to aid in the replication, fine-mapping and discovery of loci associated with inflammatory and autoimmune diseases [68]. To take advantage of the pervasive genetic overlap between many of these diseases, the Immunochip contains a dense panel of ∼130,000 SNPs located in 186 regions with known association with one or more of 12 immune-related diseases. SNPs within the regions were ascertained via dbSNP, the 1000 Genomes project (February 2010 release), and autoimmune disease resequencing projects. While not all SNPs passed the Illumina design process and made it onto the microarray, the Immunochip provides unprecedented coverage of common, low-frequency and rare variants across these 186 genomic regions. A further 50,000 SNPs that were suggestively significant in the original GWAS studies were also included. This panel served as the replication set of SNPs in Jostins et al., where over 40,000 IBD cases and controls were genotyped. The cost-effectiveness of the Immunochip (at ∼20% that of a GWAS microarray at the time) allows for studies with much larger sample sizes than GWAS and also enables powerful disease subphenotype and cross-disease comparisons [69].

## Fine-mapping associated loci

The causal variants that underlie the majority of loci discovered through GWAS remain unidentified. An associated locus will often consist of dozens of correlated SNPs in high LD spanning across many genes, with very similar association signals. In the 140 loci associated with Crohn's risk, the number of SNPs that are tagged (*r*^2^ > 0.8) by the main GWAS SNP range from 1 to 306 per locus (median 13). Narrowing these down to a single causal variant is difficult and will initially require a combination of many complementary approaches. Firstly, much larger sample sizes will be required to differentiate statistical signals at causal variants over their highly correlated neighbours. Secondly, as patterns of LD differ between different ancestral groups, obtaining samples from multiple populations can narrow the associated region for risk loci that are shared across populations. Thirdly, combining functional genetic information with association results allows variants with relevant annotations to be up-weighted in association analyses. Data from projects such as ENCODE [70] and GTEx [71] provide rich functional genomic information that can be readily integrated with GWAS results. In addition to providing functional candidates, these functional annotations can also uncover biological mechanisms through which variants act, either through the specific cell type or functional element [72–74]. Under the auspices of the IIBDGC, efforts to fine-map associated loci using the Immunochip are underway, along with transethnic studies of IBD across European and non-European populations. Ultimately, the direct modelling of these variants in cell lines and model organisms may be required for final confirmation of causality. Emerging technologies such as DNA editing through CRISPR and engineering induced pluripotent stem cells are likely to play an important role [75–77].

The *IRGM* locus exemplifies some of the challenges in identifying causal variants. The SNP initially associated with disease was later found to be in perfect LD with a 20 kb deletion upstream of *IRGM* [52,78]. This deletion was thought to be causal because it affects the expression of *IRGM*, which in turn regulates the efficiency of autophagy. A later study showed, however, that this deletion is one of several highly correlated Crohn's disease associated variants in the region that affect *IRGM* expression, none of which can reasonably be ruled out as causal [79]. Furthermore, the variants are also not associated with Crohn's disease in the Japanese population, suggesting either European-specific gene-environment interactions or the presence of an untyped causal variant that arose after the European-Asian population split [79].

## Next generation sequencing

The role of rare variants in complex diseases is currently an important area of focus in human genetics. High-throughput discovery and accurate genotyping of rare variants has recently been made feasible through large reductions in the cost of next-generation sequencing. Often cited as a possible explanation for missing heritability, rare variants are in theory likely to have much larger effect sizes than common variants due to purifying selection maintaining damaging alleles at low frequencies [61]. Indeed, loci that are associated with complex disease are enriched for rare variants that cause known Mendelian disorders and it has been suggested that recessive variants confer risk to related complex diseases when the carrier is heterozygote [80]. Independent rare variant associations are also often found in genes with known common associated variants [81–83].

Since the rare allele of individual rare variants are observed so infrequently, single variant tests of association will be underpowered for all but the most highly penetrant alleles. For instance, for an allele that doubles disease risk (OR = 2) and has a frequency of 0.1%, nearly 60,000 cases and a similar number of controls will be required for the variant to reach genome-wide significance. To increase power to detect association, rare variants are often aggregated based on characteristics such as their position within genes, functional features and allele frequencies [84]. Dozens of these burden tests have been proposed [84–87] along with methods for meta-analysis and replication [88–90].

The degree to which such variants contribute to Crohn's disease heritability is unclear, and the results from early large scale sequencing studies targeted at known susceptibility genes have been disappointing [81,91,92]. These studies typically involved sequencing the coding regions of several candidate genes in a few hundred cases and controls followed by the direct genotyping of putatively associated variants in a much larger replication cohort. Coding regions are targeted because the functional consequences of variants in these regions are much better understood than those in noncoding parts of the genome. In addition, coding variants are hypothesized to have larger effect sizes given their direct impact on protein product and are generally more evolutionarily conserved than noncoding variants [93]. Momozawa et al. [81] initially sequenced 63 candidate genes in 112 Crohn's disease cases and 112 controls with replication in an additional 288 to 928 cases and 288 to 1216 controls, and identified four independent associations in *IL23R*, although only one of these exceeded genome-wide significance. Similarly, Rivas et al. [92] sequenced 56 genes in 350 cases and 350 controls with follow-up genotyping in 16,054 cases and 17,575 controls, and identified 12 independent rare variant associations across seven genes, of which two (coding variants in *NOD2* and *CARD9*) exceeded genome-wide significance. These three genome-wide significant variants were included on the Immunochip and subsequently confirmed in Jostins et al. [59] using around 75,000 samples. However, a recent sequencing study of 25 candidate genes across 41,911 individuals, of which 3271 were Crohn's disease cases, failed to identify any novel associations [91]. A natural extension for candidate gene sequencing studies is to sequence the entire exome of cases and controls. A recent exome sequencing study in 42 Crohn's cases with follow-up genotyping in 9348 cases and 14,567 controls found suggestive rare variant associations in *PRDM1* [94]. Again, the variant failed to reach genome-wide significance and other whole exome studies with much larger sample sizes are currently underway.

The sobering results from these studies highlight the challenges in rare variant association studies. As it is currently not economically feasible to perform high coverage whole-genome sequencing in a large number of cases and controls, compromises often need to be made in terms of the number of genomic regions covered and the number of individuals. The majority of loci identified in GWAS lie in noncoding regions (Fig. 3), which have been overlooked by the current generation of sequencing studies. A large number of rare noncoding variants will play a role in gene regulation, though it remains to be seen whether their effects are large enough to be a major contributor to disease susceptibility. Performing burden tests across rare variants in regulatory regions such as promoters and enhancers may show promise. Most importantly, the sample sizes used in these sequencing studies have thus far simply been insufficient to robustly identify rare variant associations. Under certain assumptions about the effect size distribution of rare variants and selection pressures, cohorts of more than 25,000 cases may be required in order to find these signals, along with an equally large number for replication [95].

In addition to candidate gene and whole-exome sequencing, the next few years will also see the emergence of low-coverage whole-genome sequencing studies. While sequencing individuals at deep coverage (>30×) is required to obtain accurate individual genotype calls, low coverage sequencing (less than 6×) and jointly calling shared (non-private) variants across many thousands of individuals is a cost effective method for discovering rare variants. For instance, for a SNP with frequency 0.2% to be discovered, over 2000 individuals need to be sequenced at 30× coverage (60,000 genomes). In contrast, the same SNP can be identified in ∼3000 individuals sequenced at 4× (12,000 genomes) – a five-fold reduction in sequencing cost [96] and, with more sequenced individuals, greater power to detect associations. The obvious advantage of these study designs over targeted or whole-exome sequencing is that they survey the entire genome rather than individual regions. Additionally, large cohorts of sequenced individuals can be used as reference panels to impute rare variants into new and existing GWAS datasets at much greater accuracy than existing panels. Over the course of 2014–15, it is expected that over 30,000 individuals, of which ∼5000 are IBD cases, will be sequenced at low-coverage. Imputing the millions of new variants discovered from this set into ∼25,000 Crohn's disease cases (of which ∼15,000 have already been genotyped as part of GWAS) along with sufficient replication will, for the first time, enable studies with sufficient power to begin detecting associations at SNPs with frequencies in the order of 0.1–1% and ORs of 2–3 (Fig. 2).

## Genetic prediction

In addition to gaining a better understanding of disease biology, genetic information can also potentially be used for disease risk prediction. Prediction methods for complex diseases typically involve assigning a risk score to an individual based on their genotypes and previously estimated effect sizes (for instance, ORs from GWAS) across risk alleles. Risk alleles can be assigned not only based on known associations, but also include nominally associated variants. Prediction accuracy can be evaluated by methods such as the receiver operating characteristic curve (ROC), which estimates the true and false positive rates of the predictor at various risk score cut-offs [97]. The area under the ROC (AUC) is the probability that for a randomly selected pair of diseased and healthy individuals, the diseased individual will have a higher risk score. An AUC of 0.5 means that the prediction method is no better than chance, while a value of one means that the method perfectly discriminates between diseased and healthy individuals.

In Crohn's disease, genetic risk prediction is still in its infancy and does not currently offer much in terms of clinical utility. Estimates of AUC using just family history of disease, genetic risk loci or the two together range from 0.56 to 0.74 [98,99]. Including risk factors such as smoking and age into the risk model may improve the AUC. Nevertheless, given its high heritability, the theoretical maximum possible AUC assuming that all Crohn's disease risk loci have been identified and effect sizes are accurately measured is estimated to lie between 0.96 and 0.98 [100,101]. However, while this figure seems high, the utility of genetic prediction is limited given the low prevalence of Crohn's disease. Even assuming a generous disease prevalence estimate of 1% and AUC of 0.98, less than 12% of individuals who test positive (using a sensitivity cut-off of 0.93) will develop disease [100]. Increasing the threshold will increase the proportion of positively identified individuals but also exclude a higher number of cases from being identified. Genetic prediction may offer better value in existing patients through informing best course of treatment, though greater knowledge of how risk loci affect these subphenotypes will be required [102].

## Crohn's disease subphenotypes

Genetic studies have begun to shed light on loci associated with Crohn's disease subphenotypes such as disease location and clinical course. These studies often focus on one or more candidate genes with a subphenotype of interest [102–104] or can survey the whole genome in the same way as a GWAS [105]. One of the strongest signals associated with clinical outcome has been coding variants in *NOD2*, which are strongly predictive for ileal disease, stenosis, fistula and Crohn's related surgery [102]. It has been suggested that patients with these *NOD2* mutations respond poorly to bacterial antigens [106]. A noncoding variant in *FOXO3A* was also recently implicated with Crohn's disease prognosis [104]. The minor allele was found to be significantly more common in indolent patients (defined as having disease for longer than four years but with no immunomodular or intestinal resections required) than patients with frequent flaring or a complicated course of treatment (where two or more immunomodular therapies/intestinal resections were required). Despite not being associated with Crohn's disease susceptibility itself, *FOXO3A* variants are thought to affect disease outcome via regulation of IL7 and IL2 signalling pathways, whose expression patterns correlate with clinical course of autoimmune diseases [107]. The study demonstrated how integrating GWAS data into functional genomic, model organism and clinical information can uncover basic biological pathways that are associated with disease outcome.

## The genetic architecture of Crohn's disease

Putting together the results from linkage, genome-wide association and sequencing studies, the genetic architecture of Crohn's disease represents that of a typical multifactorial complex trait where a combination of multiple genes, along with the environment, lead to disease. With few exceptions, individual risk loci confer only a modest effect on disease susceptibility and together, the known loci explain ∼13% of variation in disease liability [59]. The majority of the genetic contribution to disease risk remains to be explained. Nevertheless, it is perhaps safe to say that that nearly all variants with frequency greater than 5% and ORs greater than 1.2 in individuals of European ancestry have been identified and the remaining genetic contribution will arise from a combination of common variants with ever-smaller effect sizes and rare variants [62]. In addition, all variants with large effects (OR > 3) and frequency greater than 1% have also been uncovered by GWAS and linkage studies (Fig. 1). Future sequencing studies will shed light on the exact effect size distribution of rare variants. Finally, it should be emphasised that locus discovery is not an end in itself. Challenges remain in taking what we've learned from genetic studies to build more complete models of disease pathogenesis and ultimately translating these into better patient outcomes.

**Practice points**

- Early familial observations and twin studies demonstrated that there is a significant genetic contribution to Crohn's disease risk.
- Identifying the specific genes responsible for disease will provide insights into disease biology and potential therapeutic targets.
- The discovery of NOD2 variants demonstrated the potential of linkage and candidate gene studies to identify risk loci, though subsequent efforts were largely unsuccessful.
- Since 2006, genome-wide association studies have established over 140 loci associated with Crohn's disease risk.

**Research agenda**

- Fine-mapping studies and direct experimental work will be required to identify the causal variants and biological mechanisms that underlie Crohn's disease risk loci.
- Whole-genome sequencing studies will help elucidate the contribution of rare and low-frequency variants to disease risk.
